# Remotely Conducted App-Based Intervention for Cardiovascular Disease and Diabetes Risk Awareness and Prevention: Single-Group Feasibility Trial

**DOI:** 10.2196/38469

**Published:** 2022-07-01

**Authors:** Vera Helen Buss, Marlien Varnfield, Mark Harris, Margo Barr

**Affiliations:** 1 Australian e-Health Research Centre Commonwealth Scientific and Industrial Research Organisation Herston Australia; 2 Centre for Primary Health Care and Equity University of New South Wales Sydney Australia

**Keywords:** mobile health, feasibility studies, primary prevention, cardiovascular disease, diabetes mellitus, type 2, mHealth, cardiology, heart disease, diabetes, smartphone, participate engagement, app-based intervention

## Abstract

**Background:**

Cardiovascular disease and type 2 diabetes mellitus are two of the most prevalent chronic conditions worldwide. An unhealthy lifestyle greatly contributes to someone’s risk of developing these conditions. Mobile health is an emerging technology that can help deliver health promotion interventions to the population, for example, in the form of health apps.

**Objective:**

The aim of this study was to test the feasibility of an app-based intervention for cardiovascular and diabetes risk awareness and prevention by measuring nonusage, dropout, adherence to app use, and usability of the app over 3 months.

**Methods:**

Participants were eligible if they were aged 45 years or older, resided in Australia, were free of cardiovascular disease and diabetes, were fluent in English, and owned a smartphone. In the beginning, participants received an email with instructions on how to install the app and a user guide. After 3 months, they received an email with an invitation to an end-of-study survey. The survey included questions about general smartphone use and the user version of the Mobile Application Rating Scale. We analyzed app-generated and survey data by using descriptive and inferential statistics as well as thematic analysis for open-text comments.

**Results:**

Recruitment took place between September and October 2021. Of the 46 participants who consented to the study, 20 (44%) never used the app and 15 (33%) dropped out. The median age of the app users at baseline was 62 (IQR 56-67) years. Adherence to app use, that is, using the app at least once a week over 3 months, was 17% (8/46) of the total sample and 31% (8/26) of all app users. The mean app quality rating on the user version of the Mobile Application Rating Scale was 3.5 (SD 0.6) of 5 points. The app scored the highest for the information section and the lowest for the engagement section of the scale.

**Conclusions:**

Nonusage and dropouts were too high, and the adherence was too low to consider the intervention in its current form feasible. Potential barriers that we identified include the research team not actively engaging with participants early in the study to verify that all participants could install the app, the intervention did not involve direct contact with health care professionals, and the app did not have enough interactive features.

## Introduction

Cardiovascular disease (CVD) and type 2 diabetes mellitus (T2DM) have a high prevalence worldwide, although both diseases could often be prevented through a healthier lifestyle [1,2]. From a behavioral perspective, tobacco smoking, excessive alcohol consumption, poor diet, and physical inactivity contribute greatly to the development of these conditions [2]. Technology-based behavior change interventions have the potential to promote health and prevent chronic diseases such as CVD and T2DM [3]. Commercial app stores such as Google Play Store and App Store offer a wide range of health-related apps [4]. Safavi et al [5] showed that most commercial products were not assessed for their clinical effectiveness and none were assessed for improving costs or access to clinical care. If digital health companies evaluated their products, they usually enrolled a healthy population but never assessed disease prevention as an outcome [5]. To overcome the shortcomings of the currently available health-related apps, we have developed an evidence-based and theory-based app to help people understand the risks of developing CVD and T2DM and to monitor their health behaviors. The app consists of 4 modules: a calculator for 5-year CVD and T2DM risk; goal setting and tracking functions for diet, physical activity, smoking, and alcohol intake; and an education section. A detailed description of the app can be found elsewhere [6]. We conducted usability testing on the prototype and improved the app design based on participants’ feedback. Our goal was to create an easy-to-use app without too much functionality so that it was suitable for less tech-savvy people and did not require users to own anything besides a smartphone.

Kumar et al [7] emphasized that mature intervention testing for mobile health interventions, such as larger randomized trials, should only be conducted after feasibility, usability, and preliminary efficacy have been demonstrated. In this study, we wanted to evaluate the feasibility of a remotely conducted app-based intervention. A particular problem with mobile health studies, as Eysenbach [8] pointed out, is high rates of dropout and discontinuance. Therefore, these were among the outcomes we intended to measure in the study. Furthermore, a systematic review by Donkin et al [9] showed that adherence to web-based interventions was positively associated with physical health outcomes such as physical activity, fruit and vegetable intake, and smoking. Perski et al [10] also noted that digital behavior change interventions require a certain level of user engagement to be effective. Therefore, another outcome measure for the feasibility study was adherence to app use. Perski et al [10] defined engagement with digital behavior change interventions as “(1) the extent (e.g. amount, frequency, duration, depth) of usage, and (2) a subjective experience characterised by attention, interest and affect.” This underlines that different measures might be required to determine engagement. Therefore, we collected objective data on app adherence as well as subjective data related to app usability. Overall, the objectives of this study were to evaluate the feasibility of the intervention by measuring nonusage, dropout, adherence to app use, and usability of the app over 3 months. As studies from the United States, United Kingdom, Canada, and Australia showed that mobile health users tended to be younger and female ([11-14] and Buss et al, unpublished data, 2022), a subquestion of the study was to assess whether there were any sex or age differences among the participants in terms of app usage.

## Methods

### Ethical Considerations and Informed Consent

We received ethics approval from the University of New South Wales Australia Human Research Ethics Advisory Panel G: Health, Medical, Community and Social (approval HC210520) and reciprocal approval from the Commonwealth Scientific and Industrial Research Organisation Health and Medical Human Research Ethics Committee (approval 2021_071_RR). All participants provided consent to participate in this study.

### Sample Size

Our predefined sample size was 40 participants. The number was based on a sample size calculation according to Hooper [15] and previous studies. The sample size was sufficient to determine a dropout rate of 30% to within a 95% CI of SD 7% and an adherence rate of 50% to within a 95% CI of SD 8%. We based dropout and adherence rates on data from other studies [16,17]. Other researchers recommended 24-50 participants for feasibility studies [18,19]. We used quota sampling to represent different groups within the target population. We aimed at roughly 10 participants per group (female and 45-64 years, female and ≥65 years, nonfemale and 45-64 years, nonfemale and ≥65 years). To be inclusive of nonbinary identities, we defined the sex groups as female and nonfemale assuming that about 50% of the Australian population identifies as female. We used a random number generator in Excel to select equal numbers of potential participants from each group to be invited to the study.

### Participants

People were eligible to take part in the study if they were aged 45 years and older, resided in Australia, were fluent in written and spoken English, owned a smartphone (Android or iPhone) with internet access, and had an email address. We set the start age to 45 years according to the guidelines of the Royal Australian College of General Practitioners. These state that general practitioners should screen for chronic diseases in the low-risk population and potentially initiate preventive measures starting from that age [20]. Since the intervention was for primary prevention, we excluded people who had already been diagnosed with CVD or diabetes (type 1 or 2). Participants were reimbursed for their participation with a A$30 (US $21) gift voucher.

### Intervention

We recruited participants with the help of a recruitment agency that identified and contacted potential participants from panelists. Panelist members received a link to an eligibility survey. If people fulfilled the inclusion/exclusion criteria and indicated interest in participating, we contacted them via email. After providing consent via a web-based survey, participants received another email from us that included the study instructions, the user guide, and a unique identifier. Participants were asked to download the app from the app store on their phones and then use it for 3 months (approximately 90 days). It was up to the participants how often they used the app. We only said that we encouraged regular use. For questions or technical issues, participants could get in touch with us via email. After 3 months, participants received an invitation to an end-of-study survey. The app contained 4 core modules. The first module comprised risk scores for the 5-year risk of CVD and T2DM. These were the Framingham risk score for CVD and the Australian Type 2 Diabetes Risk tool for T2DM [21,22]. The algorithms calculated the risk with information that users provided during the registration. The registration process included 21 questions. Five of these required users to enter numerical values, while the remaining questions had answer options provided. During the usability study, app testers needed less than 5 minutes to complete the process [6]. Participants could update their risk at any time. The second module was a goal-setting function. The goals were about smoking, physical activity, fruit and vegetable intake, consumption of sugary drinks, and alcohol intake. In the third module, participants could track their behavior related to these goals. They received messages to acknowledge when they achieved their self-set goals. The fourth module was for educational purposes. It included links to external websites, educational videos, and the user guide. We published a detailed description of the app elsewhere [6].

### Data Collection

We collected 2 types of data: app-generated and survey data. The outcomes we measured were nonusage rate (defined as the proportion of participants who never used the app), dropout rate (defined as the proportion of participants who completely stopped using the app at least 14 days before they received the end-of-study survey invitation), adherence rate (defined as the proportion of participants who used the app each week at least once during the 3 months), and usability of the app. For the usability assessment, we used the user version of the Mobile Application Rating Scale (uMARS), a validated instrument to measure the quality of mobile health apps [23]. The quality rating of uMARS measures the engagement, functionality, aesthetics, and information of the app. The survey contained further questions about general smartphone use. These questions were derived from a survey of the Australian Office of the eSafety Commissioner [24]. Other outcomes of interest were related to the information entered in the app and the frequency with which they were entered. Only participants who had not withdrawn from the study were asked to fill out the end-of-study survey. They received 2 reminders via email before they were considered lost to follow-up. Every participant received a unique identifier. This allowed us to control who had access to the app because even though the app was free to download from the app store, registration was only possible after entering one of the unique identifiers. We also asked participants to provide their unique identifiers at the beginning of the end-of-study survey, which allowed us to link the app data with the survey responses.

### Data Analysis

After the data collection was completed, we conducted the data analyses in RStudio using the programming language R. We used the following functions from the R Stats package. For differences between means, if data were normally distributed, we used the unpaired 2-sample *t* test (alternative hypothesis: true difference in means is not equal to 0) [25], and if data were not normally distributed, the nonparametric 2-sample Wilcoxon rank test (alternative hypothesis: true location shift is not equal to 0) [26]. For differences between categorical variables, we used Pearson chi-squared test with Yates correction for continuity [27]. We tested for correlations between variables by using Pearson product-moment correlation (alternative hypothesis: true correlation is not equal to 0) [28]. We tested for outliers by using Tukey’s rule (below: Q1 – 1.5 * IQR or above: Q3 + 1.5 * IQR) [29]. We assessed if there were differences in the outcome variables between men and women and between midaged (45-64 years) and older participants (65 years and older). We preset the significance level for all tests to .05. We gave every survey respondent a score based on their smartphone use to see whether there were differences in general smartphone use between those who used the app and those who did not (maximum score 10 points, Table 1). We thematically analyzed the free comments at the end of the survey by using a deductive approach [30].

**Table 1:** Score items for smartphone use.

Scoring items	Points
Use smartphone multiple times a day	1
Access the internet multiple times a day	1
Have mobile data on the smartphone	1
Type on the smartphone’s touchscreen without assistance	1
Use a search engine on the smartphone without assistance	1
Send an email with the smartphone without assistance	1
Take and send a picture with the smartphone without assistance	1
Install and update an app with the smartphone without assistance	1
Message or chat using internet-based apps with the smartphone without assistance	1
Make video calls with the smartphone without assistance	1

## Results

### Participants

**Figure 1:**
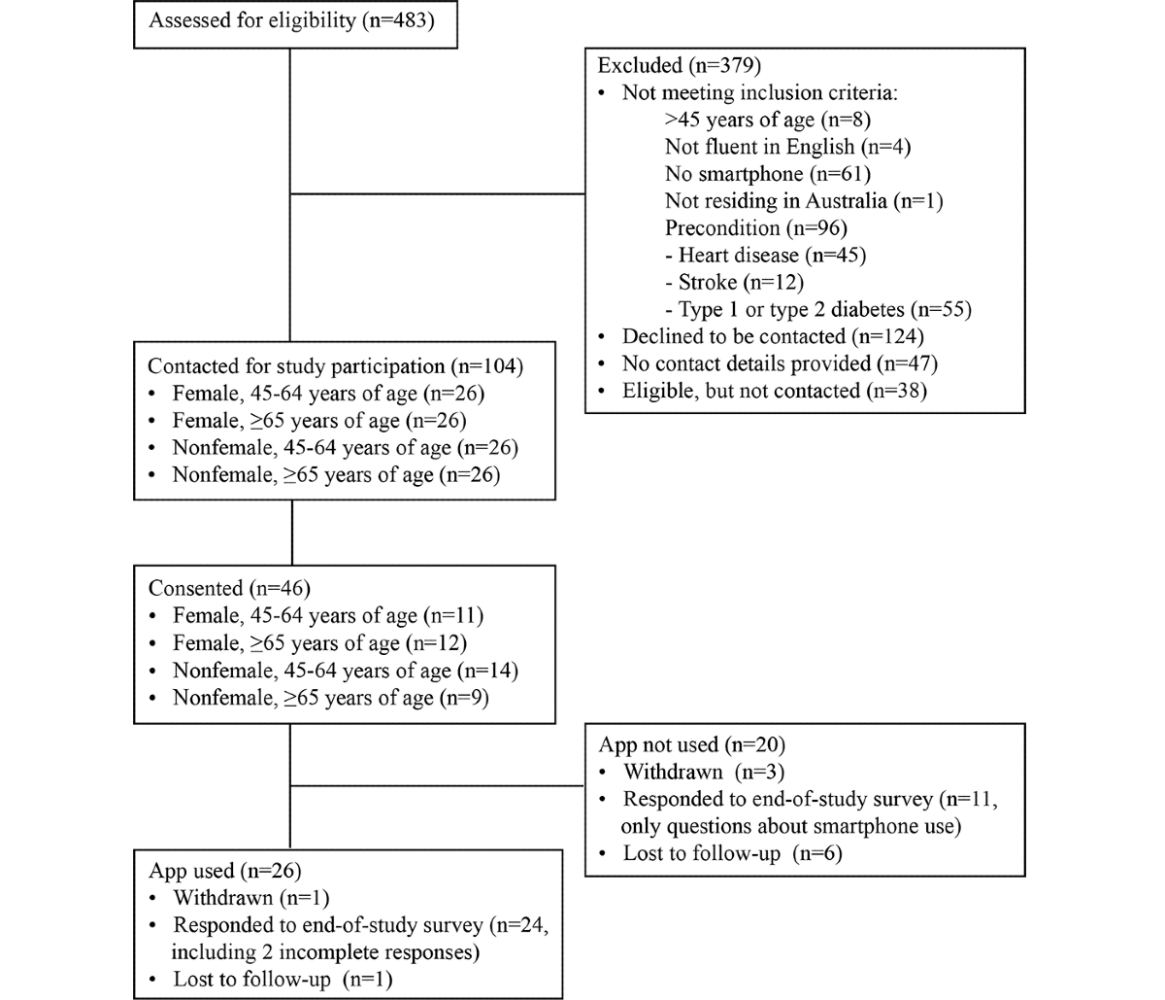
Flow diagram for this study.

This study took place between September 2021 and January 2022. We assessed 483 persons for eligibility, of which 142 persons were eligible (Figure 1). We randomly selected 26 participants for each group since this was the number of eligible individuals in the smallest group (women aged 65 years and older). Then, we invited these individuals to take part in this study, and 46 participants provided their consent. Enrolment took place between September 22, 2021 and October 14, 2021. Depending on the participants’ enrolment date, we sent out the end-of-study survey invitations on December 14, 2021 or January 5, 2022, with up to 2 reminders. Of the 46 participants, 24% (11/46) individuals were females aged 45-64 years, 26% (12/46) were females aged 65 years and older, 30% (14/46) were males aged 45-64 years, and 20% (9/46) were males aged 65 years and older. There were no statistically significant differences between the groups (χ^2^_1_=0.4; *P*=.55); none of the participants identified as nonbinary.

We received 35 end-of-study survey responses from the 46 participants at baseline regarding their general smartphone use. These consisted of 24 responses from participants who had used the app and 11 responses from participants who had not used the app (Figure 1). The 4 participants who had withdrawn from the study did not receive an invitation to the end-of-study survey. The remaining 7 participants were lost to follow-up. Most respondents (33/35, 94%) accessed the internet and used their smartphones multiple times a day and 2 respondents (6%) once a day. They stated that they connected their smartphones to the internet using a home internet connection (30/35, 86%), mobile data (31/35, 89%), public Wi-Fi (8/35, 23%), and a work internet connection (3/35, 9%). Most respondents were able to do various tasks with their smartphones without requiring assistance (Table 2). We ranked people based on their general smartphone use. The median score was 10 out of 10 points, and the minimum score for any user was 3 points. There was no statistically significant difference in the score for general smartphone use between those who used the app and those who did not (*W*=140; *P*=.75).

**Table 2:** Tasks that participants (n=35) stated that they could do with a smartphone.

Tasks	Without assistance, n (%)	Require assistance, n (%)	Never tried before, n (%)
Type on the touchscreen	32 (91)	1 (3)	2 (6)
Use a search engine	34 (97)	1 (3)	0 (0)
Send an email	33 (94)	2 (6)	0 (0)
Take and send a picture	33 (94)	2 (6)	0 (0)
Install and update an app	32 (91)	2 (6)	1 (3)
Message or chat using internet-based apps	30 (86)	1 (3)	4 (11)
Make video calls	28 (80)	0 (0)	7 (20)

### Nonusage, Dropout, and Adherence to App Use

The nonusage rate was 44% (20/46). Of the 26 participants who used the app, 16 participants were 45-64 years old and 10 were 65 years or older. There was no statistically significant difference between app use and age groups (*χ*^2^_1_=0.7; *P*=.41), but there was a statistically significant difference between sex and app use (*χ*^2^_1_=7.2; *P*=.007), with more men (18/26) using the app than women (8/26). The median age of app users at baseline was 62 (IQR 56-67) years. The oldest app user was 73 years old, and the youngest was 47 years old. Table 3 shows further characteristics of the app users at baseline and the duration of app use. The dropout rate was 33% (15/46). Eight participants used the app at least once a week. That represents an adherence rate of 17% (8/46) of the total sample and 31% (8/26) of all app users. The median time between the first and last app use was 54 (IQR 4-83) days. Owing to small differences between enrolment and survey completion dates, the maximum potential time for a participant to be included in the study varied slightly. There were no statistically significant correlations between age and duration of app use (*t_24_*=–0.84; *P*=.41; *r*=–0.168; 95% CI –0.522 to 0.234). Neither was there a statistically significant difference between sex and duration of app use (*W*=65; *P*=.72).

**Table 3:** Baseline characteristics of app users (n=26) and their duration of app use.

Characteristics of app users			Values, n (%)
**Demographics**			
	Female	8 (31)	
	Age ≥65 years	10 (39)	
	Born in Australia	17 (65)	
**Cardiovascular risk**			
	Low	16 (64)	
	Moderate	4 (16)	
	High	5 (20)	
**Diabetes risk**			
	Low	0 (0)	
	Moderate	7 (27)	
	High	19 (73)	
	Regular smoker	7 (27)	
**Healthy lifestyle**			
	Physical activity, 2.5 hours per week	18 (69)	
	Daily fruit and vegetable intake	19 (73)	
**Duration of app use**			
	1 day	5 (19)	
	2-7 days	2 (8)	
	8-30 days	3 (12)	
	31-60 days	3 (12)	
	61-90 days	13 (50)	

App users calculated their CVD and T2DM risk in a median twice (IQR 1-4), with a maximum of 14 times. For some app users, the risk changed over time, but only on 4 occasions this led to a different risk category displayed in the app. After the registration, app users were automatically directed to the goal-setting module. They set goals of a median of once (IQR 1-3) and a maximum of 11 times, which was an outlier. Six app users never set a goal, and 3 never tracked health-related behaviors. The median number of times app users tracked health-related behaviors was 14 (IQR 1-57), with a maximum of 137 times. This value was not an outlier. Among those (15/26, 58%) who tracked their health behaviors on at least 7 days, 12 persons (80%) tracked them on a median every day, 2 persons (13%) on a median every second day, and 1 person (7%) on a median every third day. Among those who regularly tracked health-related behaviors, 4 people (33%) never reached their goals for all health-related behaviors in 1 day. The maximum was reached by 1 person who achieved their goals in 8 days. This corresponded to 13% (8/61) of the days that the person recorded health-related behaviors. The health-related behavior that app users achieved the least was minutes of physical activity per week.

### Usability of the App

**Figure 2:**
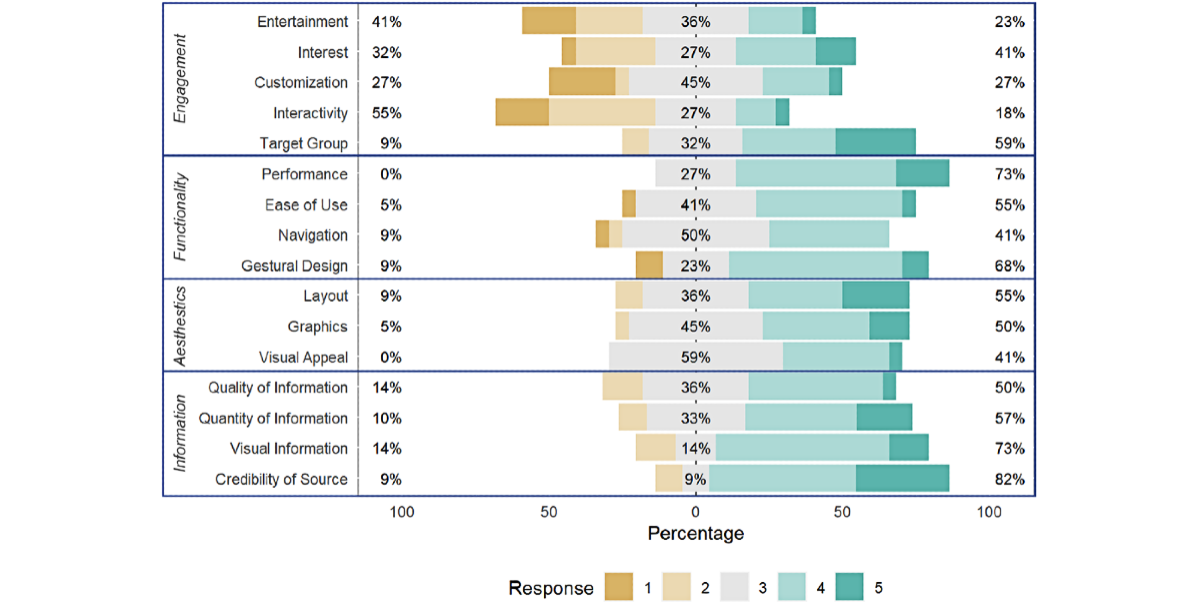
Results of the app quality rating on the Likert scale in the user version of the Mobile Application Rating Scale (n=22).

The results from the uMARS are based on 22 participants who had used the app and had completed the end-of-study survey in its entirety. The overall app quality rating on the uMARS was 3.5 (SD 0.6) points out of a maximum of 5 points. Figure 2 shows the responses of the uMARS app quality rating on a 5-point Likert scale for each item. The highest score was for information with a mean of 3.70 (SD 0.67) points, followed by aesthetics (3.58 [SD 0.65]), functionality (3.57 [SD 0.56]), and engagement (2.99 [SD 0.86]).

**Figure 3:**
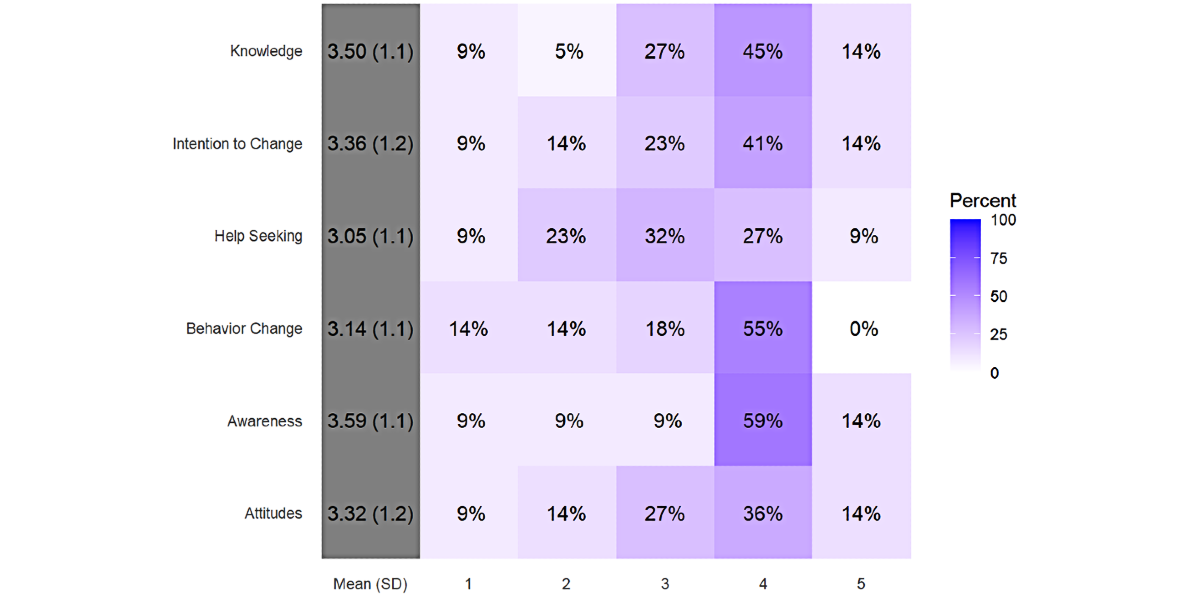
Results of the perceived impact rating on the Likert scale.

Regarding the subjective quality of the app, of the 22 users, 2 (9%) app users stated that they would recommend the app to everyone, 3 (14%) would recommend it to many people, 3 (14%) would recommend it to several people, 9 (41%) would recommend it to very few people, and 5 (23%) would not recommend it to anyone. Of those 8 app users who would recommend the app to everyone, many people, or several people, 6 (75%) were 45-64 years old, 2 (25%) were 65 years or older, 6 (75%) were males, and 2 (25%) were females. They rated the app quality with a mean score of 4.07 (SD 0.41). Among the 14 app users who would recommend the app to only very few or none, 8 (57%) were 45-64 years old, 6 (43%) were 65 years and older, 12 (86%) were males, and 2 (14%) were females. They provided a mean app quality score of 3.11 (SD 0.32). The difference in the mean scores was the greatest for engagement (3.78 [SD 0.65] vs 2.54 [SD 0.61]), followed by aesthetics (4.17 [SD 0.59] vs 3.24 [SD 0.40]), information (4.25 [SD 0.42] vs 3.38 [SD 0.32]), and functionality (4.09 [SD 0.40] vs 3.27 [SD 0.39]). When asked how often they think they would use the app in the next 12 months, 10 (46%) app users answered never, 1 (5%) answered once or twice, 3 (14%) answered 3-10 times, 6 (27%) answered 10-50 times, and 2 (9%) answered more than 50 times. When asked about payment, 14 (64%) app users responded that they would definitely not pay for the app, 4 (18%) responded probably not, 3 (14%) responded they might or might not, and 1 (5%) responded probably yes. The last set of uMARS questions was about the perceived impact on the users’ knowledge, attitudes, and intentions related to the target health behavior. Responses were based on the 5-point Likert scale (Figure 3). All mean values were between 3.0 and 4.0. The highest score was for awareness (mean 3.6 [SD 1.1]), with 73% (16/22) of the app users somewhat or strongly agreeing that the app had increased their awareness of the importance of addressing the health behaviors. The lowest score was for help-seeking (mean 3.1 [SD 1.1]), which asked whether participants agreed that the app would encourage them to seek further help to address the health behavior (if needed).

Among the app users, 15 people left comments about the app at the end of the survey. We identified 6 themes (issues with self-monitoring, lack of interaction, credibility, user-friendliness, interaction with health care professionals, and privacy). One of the main themes we identified was issues with self-monitoring of health-related behavior. Some app users could not see the health-related behavior trends shown over time. One described initial confusion over the difference between daily and weekly goals when entering the number of alcoholic drinks consumed. Others mentioned that it took them too long to enter the values manually. One said it would have been nice to link the app to a step counter app on the phone. Some participants would have liked reminders for self-monitoring. This also relates to the theme of lack of interaction between users and the app. One specifically stated that the app lacked features that incentivize app use. A further theme was the credibility of the information. One person found the app inaccurate because it only considered waist circumference but not BMI. However, others mentioned that they liked the health information provided and found it credible. Regarding the user-friendliness of the app, some found the app clunky, while others specifically said that they felt it was easy to use. Concerning interaction with health care professionals, one person explained that using the app encouraged the person to get blood glucose levels checked and make an appointment with a cardiologist. Another person outlined that they were already working with their general practitioner on the health behaviors targeted with the app due to increased disease risk and reported using a diet-tracking app. One person raised privacy concerns and suggested that to protect their privacy, a password should be included to safeguard their information from other people who might be using their smartphones. One person who did not use the app said they could not access it. Other participants (10/46, 22%) had reached out to us via email at the beginning of the study to receive help downloading the app and registering.

## Discussion

### Principal Results

Our objectives were to evaluate nonusage, dropout, adherence to app use, and usability of the app-based intervention for cardiovascular and diabetes risk awareness and prevention over 3 months. The nonusage and dropout rates were high, and the adherence rate was low. The overall quality rating on the uMARS was satisfactory. However, scores for interactivity and entertainment, which are part of the engagement section, were particularly low. We noticed differences between those who would recommend the app to everyone, many, or several people and those who would recommend it to only very few people or no one. Interestingly, the difference in the mean scores was the smallest for app functionality. Since our sample size of app users was quite small, one must interpret these differences cautiously.

Our results showed issues with the adoption of and engagement with the app-based intervention. We have different hypotheses about what might have contributed to these issues. Possible explanations for nonadoption are (1) problems installing the app, as stated by a participant in the survey; (2) the use of other health apps that better suit their needs and preferences, as mentioned by 2 participants in the survey; and (3) other pressures such as those caused by the COVID-19 pandemic with people potentially being more concerned with them or a family member contracting COVID-19 than developing CVD or T2DM. A likely explanation for the low engagement is that the app lacked interactive features. Although the app included 2 types of push notifications when users achieved their goals, the data analysis showed that participants barely met the required conditions to see these messages. That means participants received little to no notifications through the app. Although the registration process required app users to answer a total of 21 questions, we saw no indication that this affected adherence. Since each participant had to enter a unique identifier at the beginning of the registration process before proceeding to the questions, we could determine that all app users completed the registration. The non–app users never saw the questions. Participants did not indicate that they perceived the risk scores as disempowering or that they were overwhelmed by 2 conditions being integrated into the app. Even though the Framingham CVD risk score does not directly rely on data about physical activity and diet [21], the app did not show users exactly how the risk was calculated unless they used the link to the external source.

### Limitations

We estimated that a sample size of 40 participants would be sufficient to detect a 30% dropout and 50% adherence rate. We did not factor in nonusage when calculating the sample size because we did not anticipate nonusage to be an issue. When analyzing the data, we decided to differentiate between nonusage and discontinuation of use, that is, dropout. In retrospect, a larger sample size could have been beneficial. However, the sample size was sufficient to answer our research question. This study showed that asking people aged 45 years and older to download the app and expect them to use it over 3 months without additional interaction was not feasible. In addition to the small sample size, another limitation of this study was that we recruited participants through a recruitment agency. We noticed that some participants had completed the end-of-study questionnaire in full, including the question from the uMARS, even though they did not use the app. Those answers were excluded from the analysis. A further limitation of this study was that we did not collect data about the participants’ educational level or socioeconomic status. Another factor that may have influenced the study is that it took place during the COVID-19 pandemic, and some participants might have been in a government-regulated lockdown. It might partly explain the high nonuse and dropout as well as the low level of engagement, as participants may have had other health priorities on their minds. However, others such as Wright et al [31] have pointed out that the COVID-19 pandemic had led to decreased screening and prevention service rates for chronic diseases, which underscores the value of this app-based intervention.

### Comparison With Prior Work

In comparison to the findings in our study, Krishnamurthi et al [32] reported that recruitment for their app-based study for stroke risk assessment was feasible and that the app had reasonable acceptance. However, more participants in the app intervention group dropped out (7/26) than those in the control group (1/24) [32]. Krishnamurthi et al [32] also found that owning a smartphone did not automatically mean that the owner could download and use the study app. In a preventive CVD intervention in collaboration with the patients’ general practitioners and including a web application, Coorey et al [33] reported that participants logged in, on average, 18 times within 12 months, most frequently to check the goal tracking/progress module and least frequently to use the chat function. In this study, too, participants rarely used the web application [33]. In contrast to the app in our study, participants had the option to receive heart health advice, motivational messages, and reminders via email or text message to support the computer-user interaction [33]. About half of those who signed up found the messages helpful [33]. As opposed to our study findings, Lavikainen et al [34] found in their app-based study for T2DM prevention that older people were more likely to engage with the app [34]. The authors reported that only those who intensely interacted with the app achieved noteworthy changes in lifestyle-related risk factors. These active users were more likely to have already had a better diet, higher levels of physical activity, and lower stress levels at baseline [34]. Leung et al [35], in their study of an app-based intervention for T2DM risk, showed that app users who received a high risk of developing T2DM significantly improved their daily vegetable intake and physical activity over 2 years but not their smoking behavior or alcohol consumption. In our study, participants ranked the credibility of sources particularly high on the uMARS. We included links to the sources for the risk scores because, during usability testing, we noticed that users wanted to know more about the scores used in the app. In contrast, Fijacko et al [36] found in their systematic review of 3 major app stores that only 9 out of 31 apps intended for T2DM risk calculation disclosed the name of the risk score they had implemented in the app.

Several studies focusing on weight loss reported differences in self-monitoring of diet, physical activity, and body weight by using apps. For example, Carpenter et al [37] found that consistency with an app-based intervention over 6 months was higher for self-monitoring physical activity. At the same time, disengagement was higher for self-monitoring of weight and diet intake. Participants who additionally received a face-to-face interventional component had better outcomes for consistency and disengagement for self-monitoring of dietary intake. Interestingly, greater consistency and longer time to disengagement for self-monitoring of diet and weight led to greater weight loss, but this was not the case for self-monitoring of physical activity [37]. Turner-McGrievy et al [38] stated that participants who had to enter dietary intake manually were more likely to form the habit of self-monitoring than those who used lower burden options (wearable bite-counter device or photo-based app). Further, Butryn et al [39] detected better adherence to self-monitoring over time for physical activity, which was tracked via sensors instead of diet and weight, which were tracked via a food diary in the app and a wireless body weight scale. Although these findings are not specifically associated with CVD or T2DM risk, we think they still have a relevant implication for our study: adherence to self-monitoring does not necessarily mean that users would achieve behavioral goals.

Carpenter et al [37] also argued that even though automated tracking is more convenient for app users, it might not achieve the anticipated behavior change. Some app users suggested automated tracking of physical activity to increase engagement with our app. We believe that it is likely to increase the frequency of tracking but not necessarily the achievement of physical activity goals or even disease risk reduction. We did not include automatic physical activity tracking in the app because of privacy and equity issues. As mentioned by a participant, it would be possible to collect daily step counts by linking the app to another app such as Samsung Health, Google Fit, or Apple’s Health app. However, that would require data-sharing permissions with third-party providers. However, we decided against implementing wrist-worn devices in the intervention because we did not want to disadvantage people who cannot afford wearable devices. Montgomery et al [40] showed that these are legitimate concerns. However, we could provide users with the option to link the app to another app, a physical activity tracker, or a smartwatch, ensuring that they were aware of the data-sharing permissions and keeping the option to enter data manually.

### Implications and Future Work

This study demonstrated that it would not be feasible to implement the app-based intervention in the current form because we would not expect sufficient engagement with the app to achieve significant behavior change in participants. There are different options on how we could adjust the intervention to hopefully achieve fewer nonusage and dropouts as well as higher adherence. One option would be to check in with participants at the beginning of the study to ensure that they could download the app. Potentially, that could significantly reduce the number of people who never used the app. Another option is to increase the number of interactive features in the app so that app users feel more motivated to use the app regularly. We could also enable voice input options to facilitate data entry. However, that would require access to the phone’s audio input, which may risk the user’s privacy. Additionally, it would increase app-specific storage. Further, we could include interactions with health care professionals in the intervention to improve adherence. We considered this when developing the intervention. However, the evidence for its superiority was inconclusive, for example, as reported by Cucciniello et al [41] in their systematic review of mobile apps for chronic disease management. We saw a potential advantage for primary prevention by directly approaching health consumers because preventative measures traditionally leave out certain population groups because they do not visit a general practitioner. Feng et al [42] found that Australians with multiple lifestyle-related risk factors are among the least likely to see their general practitioner. Hence, they argued that preventative interventions should also be offered outside the traditional health care setting.

Byambasuren et al [43] reported that people found a recommendation by their general practitioner to be a facilitator for the uptake of mobile health apps. Similarly, Nguyen et al [44] explained that general practitioners could be involved in mobile health interventions through app promotion and regular review of patient-centered app data. They explained that general practitioners could review the data during the medical consultation or remotely via a web-based portal [44]. Nguyen et al [44] pointed out that an issue with this approach is that general practitioners in Australia are typically time-poor. It would not be easy to fit the data review into their schedule. The same was confirmed by general practitioners in the study by Coorey et al [33]. Therefore, Nguyen et al [44] proposed involving allied health professionals such as nurse practitioners or community pharmacists. In the next step, we could implement the discussed options and test them in another feasibility study before considering an implementation study to assess the intervention’s effectiveness.

### Conclusions

The app-based intervention proved to be unfeasible in its current form because too many study participants never used the app or dropped out and too few used the app weekly. We identified potential barriers such as no active query from the research team at the start of the study as to whether participants were able to install the app, insufficient interactive app features, as well as no direct interaction with health care professionals. We believe it was important to conduct this feasibility study before evaluating the intervention’s effectiveness in a larger trial. It saved resources for a study that likely would not have shown intervention effectiveness owing to low user engagement.

## Abbreviations

CVD: cardiovascular disease
T2DM: type 2 diabetes mellitus
uMARS: user version of the Mobile Application Rating Scale
